# SWOT analysis of decentralised clinical trials from an ethical, legal, regulatory and operational perspective

**DOI:** 10.1186/s12910-026-01391-w

**Published:** 2026-02-09

**Authors:** C Murciano-Gamborino, L Pérez-Breva, AJ de Jong, GJM van Thiel, Y Santa-Ana-Tellez, M Boeckhout, SJ Siiskonen, T van Rijssel, H Gardarsdottir, J Fons-Martinez

**Affiliations:** 1 Vaccines Research Area, Foundation for the Promotion of Health and Biomedical Research in the Valencian Region (FISABIO - Public Health), Valencia, Spain; 2 CIBER de Epidemiología y Salud Pública, Instituto de Salud Carlos III, Madrid, Spain; 3 Julius Center for Health Sciences and Primary Care, University Medical Center Utrecht, Utrecht, The Netherlands; 4 Division of Pharmacoepidemiology and Clinical Pharmacology, Utrecht Institute for Pharmaceutical Sciences, Utrecht University, Utrecht, The Netherlands; 5 Rotterdam University of Applied Sciences, Rotterdam, The Netherlands; 6 Department of Clinical Pharmacy, Division Laboratory and Pharmacy, University Medical Center Utrecht, Utrecht, The Netherlands; 7 Faculty of Pharmaceutical Sciences, University of Iceland, Reykjavik, Iceland

**Keywords:** Decentralised clinical trials, Ethics, SWOT analysis, Clinical trials, Regulation, Telemedicine, EHealth

## Abstract

**Background:**

Decentralised clinical trial (DCT) approaches are characterised by moving operational procedures from the traditional on-site setting to the participant’s immediate surroundings through the use of digital tools. DCTs have received increased interest in recent years. In this article, a SWOT analysis was carried out to identify the strengths, weaknesses, opportunities, and threats for DCT approaches from an ethical, legal, regulatory and operational perspective, as compared to conventional clinical trials (CCTs).

**Methods:**

During a two-day workshop organised on 5 and 6 April 2022 at Utrecht University, a group of 10 experts from the IMI project Trials@Home identified 8 key trial activities (KTAs) which operational procedures differ between DCTs and CCTs and carried out a SWOT analysis for each activity separately: implementation of decentralised/remote (electronic) informed consent; decentralised screening of potential trial participants; home health visits; telemedicine visits; participant’s self-monitoring; delivery and return of investigational product; clinical trial oversight; and remote safety monitoring. These analyses were categorised to form an overall SWOT analysis for DCTs.

**Results:**

The analysis revealed 48 strengths, 62 weaknesses, 5 opportunities, and 13 threats across the eight KTAs. Categorisation distilled these into 11 strengths, 13 weaknesses, 2 opportunities, and 4 threats for DCTs. The use of digital technologies, lack of face-to-face contact and harmonisation of regulation were the most targeted issues.

**Conclusions:**

DCTs offer potential advantages, such as remote participation, greater autonomy in decision-making, simplified monitoring and access to diverse populations. However, challenges include digital technology barriers, reduced face-to-face contact, difficulties in identity verification and privacy issues. Addressing these challenges requires adequate training of participants and healthcare professionals, robust identity verification methods, strong security measures, and adaptation of communication and procedures. A hybrid approach combining traditional and decentralised elements seems feasible at this stage. Future research should explore the identified strengths and weaknesses of DCTs, propose ethical solutions to remaining challenges and ensure the inclusion of diverse stakeholders.

**Supplementary Information:**

The online version contains supplementary material available at 10.1186/s12910-026-01391-w.

## Background

In recent years, clinical trials (CT) have undergone a significant transformation driven by technological advances and the need to adapt to new circumstances, such as the COVID-19 pandemic [1, 2]. This change has been reflected in particular in innovative Decentralised Clinical Trials (DCT) approaches that allow the trial participants to participate in clinical trials from their homes or from their immediate surroundings, minimising or eliminating the need to travel to research sites. The decentralisation can be complete or partial (hybrid CT), depending on whether traditional trial procedures are entirely or partially maintained [3]. Santa-Ana-Tellez et al. [4] define Decentralised clinical trials as “*an operational model of clinical trials in which trial activities are designed to take place at*,* or in the vicinity of*,* the participant’s home*,* rather than at a traditional clinical site. This approach can make use of technologies and other innovative operational approaches to facilitate data collection*”. On the other hand, Covaros et al. [5] indicate that two key elements determine the level of decentralisation in a clinical trial: the location where data are captured (at a centre or near the participant’s environment) and the method of data capture. Data can be captured manually or digitally, through an intermediary or directly and passively (automatically) or actively.

This new strategy of conducting CTs has been motivated by the search for solutions to the traditional challenges of recruitment, retention, diversity and geographic accessibility.

However, such a paradigm shift is not without significant challenges. The implementation of remote electronic consent, decentralised screening, the volume and quality of data generated, the distribution of roles and responsibilities, and remote safety monitoring are critical issues that need to be addressed.

To better understand the implementation and impact of DCT approaches, it is essential to explore the shift from traditional clinical trials to decentralised strategies. In this context, the importance of regulation and specific guidelines orienting the conduct of DCTs is highlighted. The Clinical Trials Transformation Initiative (CTTI) [6] in the United States or European Innovative Medicines Initiative (IMI) Trials@Home [7] project in Europe have played a crucial role in exploring opportunities and challenges, as well as developing pilot programmes to assess the feasibility and to compare the scientific and operational quality of traditional and decentralised approaches.

Additionally, the European Commission, the European Medicines Agency (EMA), the Heads of Medicines Agencies (HMA), and the U.S. Food and drug Administration (FDA) have issued specific recommendations on decentralised elements in clinical trials [8, 9], recognising the importance of establishing clear rules and procedures to guide the implementation and development of DCTs.

The aim of this article is to describe the results of a SWOT analysis (Strengths, Weaknesses, Opportunities, and Threats) of DCTs from ethical, legal, regulatory and operational perspective. Based on the results, we aim to clarify some of the aspects of decentralised approaches and highlight key aspects to be taken into account in the design of future DCT approaches.

## Methods

### Study design

A qualitative study was conducted to identify key trial activities (KTA) that differ between DCTs and conventional clinical trials (CCTs). These activities were identified through a multidisciplinary discussion informed by regulatory guidance on clinical trial management during the COVID-19 pandemic and previous work conducted within the Trials@Home project [3, 10–23]. Once the KTAs were identified, a SWOT analysis was conducted for each of them, and finally a categorisation process was used to identify the components of the overall SWOT matrix of the DCTs.

### Description of the participants

The group was composed of 10 multidisciplinary experts^1^ (7 women, 3 men) with different backgrounds: clinical trials coordinator, ethicist and legal experts, medical doctor, pharmacists, pharmacoepidemiologists and sociologist. The experts were members of the Trials@Home work package 4 (EAGLE – Ethics, regulAtion, Gcp, LEgal and data privacy aspects). These experts are largely overlap the authors of this paper.

### Data collection and analysis

Data were collected during a two-day workshop held on 5 and 6 April 2022 at Utrecht University. On the first day, starting from the definitions and activities comprising each Basic Building Block (BBB)—input obtained from previous work within the Trials@Home project—experts identified the key topics relevant for this SWOT analysis study and, from these, defined the key trial activities (KTAs) using moderated focus groups structured around BBB framework [12], focusing on processes that raise specific ethical, legal, regulatory, or operational considerations and whose procedures differ between decentralised clinical trials (DCTs) and conventional clinical trials (CCTs). All contributions were captured in real time in detailed notes and a shared Word file projected during the discussion, which together constituted the qualitative data corpus.

A thematic analysis approach was applied during the workshop itself through these specific, reproducible steps:

- Step 1 - Identification of key topics: Moderators read aloud the activities comprising each BBB and assigned an initial descriptive label in the Word file capturing the key themes from an ethical, legal, regulatory, and operational perspective (e.g., "informed consent process", "IMP delivery", "follow-up visit"). Similar or overlapping labels were grouped under provisional key topics, creating new key topics when ideas did not fit existing categories.
- Step 2 - Development and review of KTAs: Moderators read aloud the list of key topics and participants identified the activities deriving from each key topic that differed between DCTs and CCTs. Moderators periodically paused to review the list of activities with participants, refining categories by splitting, merging, or renaming as necessary to define candidate KTAs.
- Step 3 - Consensus building: Participants systematically reviewed each BBB domain, its key topics, and emerging KTAs to confirm the final set and the specific activities included under each KTA; discrepancies were discussed until explicit agreement was reached. The final list was recorded in the Word file and summarised in Table 1.

**Table 1:** Thematic identification of KTAs based on BBBs

BBB	Key Topic	KTAs
Operational and coordination	The elaboration of informed consent	• Implementation of decentralised/remote (electronic) consent • Clinical trial oversight • Decentralised screening of potential trial participant
Setup & Design		
Recruitment & Enrolment	Obtaining informed consent The possibility of screening visits out of the site	
Patient Engagement		
Intervention and Follow Up	Shipping medication to a participant’ home	• Delivery and return of investigational product
	The possibility of follow-up visits out of the site	• Telemedicine Visits • Home health visits
Data Acquisition & Processing	Trial monitoring with remote verification of source data	• Participant’s self-Monitoring • Remote safety monitoring
Close Out & Reporting		

On the second day of the workshop, the same group of experts used the agreed KTAs as a basis to identify and agree on the items to include in a SWOT matrix (strengths, weaknesses, opportunities, threats) for each KTA, guided by predefined questions distinguishing internal versus external factors and positive versus negative aspects (Table 2). Proposed items were discussed, clarified, and where appropriate merged with related items, such that the thematic grouping and refinement of ideas for each SWOT quadrant also took place within the workshop until consensus was achieved; the resulting KTA-specific SWOT matrices were documented in the shared Word file.

**Table 2:** Questions to identify the elements of the SWOT matrices

Internal factors	Positive aspects	Negative aspects
	Strengths: What aspects of DCTs facilitate or enhance compliance with ethical, regulatory, and legal requirements compared to CCTs?	Weaknesses: What aspects of DCTs hinder or worsen compliance with ethical, regulatory and legal requirements compared to CCTs?
External factors	*Opportunities*: How do ethical, regulatory and/or legal factors enhance or facilitate the implementation of DCT?	*Threats*: How do ethical, regulatory and/or legal factors limit or hinder the implementation of DCT?

In the days following the workshop, notes and audio recordings were reviewed to refine and expand the brief labels into full narrative descriptions of each KTA. These descriptions were circulated to all participants and iteratively revised until consensus was reached on their wording and accuracy, ensuring they faithfully reflected the workshop discussions (see descriptions in additional file 1).

Likewise, all items from the KTA-specific SWOT matrices were collated in a single Excel file and grouped into broader cross-cutting categories to construct the overall SWOT matrix for decentralised clinical trials. Overlapping or closely related items were merged, checked against the workshop notes for consistency, and organised into higher-level themes, resulting in an overall SWOT matrix comprising 11 strengths, 13 weaknesses, 2 opportunities, and 4 threats. This final consolidation step did not introduce new themes beyond those developed through the in-workshop thematic analysis but rather provided a more abstract synthesis of the workshop findings.

## Results

8 KTA were identified and defined: implementation of decentralised/remote (electronic) consent; decentralised screening of potential trial participant; home health visits; telemedicine visits; self-monitoring; delivery and return of investigational product; clinical trial oversight and remote safety monitoring.

A total of 128 items (48 strengths, 62 weaknesses, 5 opportunities and 13 threats) were detected in the SWOT matrices for each of the KTAs, whose distribution among the different KTAs is shown in Table 3.

**Table 3:** Number of items in each SWOT analyses matrix

KTA1: Implementation of decentralised/remote (electronic) consent	S	W	O	T
	16	11	3	2
KTA2: Decentralised screening of potential trial participant	8	12	2	0
KTA3: Home health visits	5	10	0	4
KTA4: Telemedicine visits	8	11	0	2
KTA5: Participant´s self-monitoring	8	7	0	3
KTA6: Delivery and return of investigational product	1	5	0	1
KTA7: Clinical trial oversight	0	4	0	1
KTA8: Remote safety monitoring	2	2	0	0
Total KTAs	48	62	5	13

*S* Strengths, *W* Weaknessess, *O* Opportunities, *T *Threats

The SWOT matrices for each of the 8 identified KTAs are presented in narrative form below (the items comprising each matrix can be found in additional file 2).

### KTA1. Implementation of decentralised/remote (electronic) consent

Strengths of remote (electronic) consent include improved recruitment and retention of clinical trial participants by ensuring that the implications of their participation in the trial are understood. Remote accessibility also promotes diversity of the representative population. By accommodating different literacy levels and incorporating explanatory videos and audio material, electronic consent promotes greater health literacy. It simplifies complex medical concepts through layman’s language and broadens access to information via a layered approach.

Implementing electronic consent can improve the participants’ experience, for example, by including interactive questionnaires that facilitate discussions on informed consent and verify participants’ understanding. It also simplifies access to updated information when changes occur in consent documents.

However, this approach also has certain weaknesses. The costs associated with developing electronic consent can be substantial, and the exclusion of digitally illiterate participants or those without access to devices raises ethical and equity concerns about access to clinical research. In addition, the lack of in-person interactions might prevent the establishment of trust between participants and researchers, as well as hinder verification and privacy of interlocutors (participants and their carers). It may end up distorting the informed consent process by focusing only on the content of the informed consent materials and reducing explanation and discussion with the researcher to a minimum or even eliminating it. Also, the absence of paper backups can pose risks in terms of security and compliance. The use of the layered approach may lead to information not included in the main layer because it is being perceived as unimportant and omitted.

The main opportunity is the harmonisation of regulations related to remote consent and the authorisation of electronic signatures. The main threats identified referred on the one hand to the development of trusted applications for the verification of the identity of the persons involved and of electronic signatures, and on the other hand to the legislative differences existing between countries at the time this research was carried out.

### KTA2. Decentralised screening of potential trial participants

Among the strengths identified, the ease of scheduling the visit stands out, allowing for more efficient time management for participants and researchers. Additionally, conducting the screening in the participant’s environment can facilitate their participation by making them feel more comfortable and, in the case of home visits, would allow information to be collected from the participant’s environment that would not otherwise be captured (e.g. relating to the person’s living conditions). Furthermore, the possibility of recording the examinations performed during the screening process can improve the assessment of participants’ eligibility. Decentralised screening may be particularly favourable for participants who find it difficult to travel (e.g. people with reduced mobility, strict work schedules), as it avoids the need to travel to the research site.

However, this approach also has important weaknesses. For example, poor management of screening recordings may raise privacy and security concerns, and the possibility of incorrect measurements made by patients themselves may compromise the integrity of the data collected. In addition, if screening takes place via teleconference, the lack of a complete physical examination and the inability to verify underlying conditions that could affect a participant’s suitability for the trial (e.g. alcoholism, physical characteristics not visible on video) may create risks to participants and to the accuracy and validity of the results. As mentioned above, the remote performance of this activity can lead to misgivings on the part of both the participant and the researcher. That is, when the nurse/investigator and the potential participant are at different locations and screening is based on a teleconference the identity of either party can be more difficult to verify. Lack of trust can make the participant feel uncomfortable about recording their physical examination.

There are also opportunities to improve this process, such as increasing the autonomy of participants by offering flexibility. For example, physical examinations could be performed by local doctors, which could address some of the current limitations and improve the acceptance of and participation in DCTs.

Finally, it is important to consider the potential threat**s** associated with decentralised participant selection, although no specific threats have been identified in this study.

### KTA3. Home health visits

The strengths we identified in home health visits mainly relate to the convenience they provide for certain participants, particularly those with mobility limitations or vulnerable groups such as the elderly or children. A significant advantage is the reduction in the travel burden, which can improve accessibility and participation in the study. Additionally, home visits offer greater flexibility, as in some cases they can occur in various places within the participant’s environment, not just their home. The staff’s visit to the participant’s surroundings can also provide a more complete picture of their situation, allowing for the detection of external factors that could affect their health and well-being.

However, home health visits may also be impacted by certain weaknesses. For example, there is a risk of deterioration or contamination of samples during sample collection or transport. Receiving health care staff at home may cause stress for participants and their household members or home visits may be perceived as invasive or stigmatising (e.g. neighbours may notice the visit), and some activities may be difficult or even unfeasible to conduct at home.

In addition, there are some structural problems related to the health personnel who carry out these visits, such as differences in qualifications or competences between countries or regions or the lack of professionals with a suitable professional profile. Also, outsourcing this service to external parties may complicate the supervision of activities or create disagreements with investigators and may transfer the burden of visits from trial participants to research staff.

No specific opportunities were identified. A threat, identified in relation to conducting multi-centre trials, could be that differences in local regulations and levels of professional certification affect the consistency and quality of home health visits. In addition, the shortage of qualified health professionals available to carry out these visits has also been identified as a threat (as well as a weakness) and represents a major challenge, especially in areas with limited resources or in trials that require a large number of home visits or where the population is widely dispersed.

## KTA4. Telemedicine visits

Telemedicine visits, as an integral part of DCTs, have a number of strengths that influence their effectiveness and efficiency. One of the main advantages lies in the flexibility they offer in terms of scheduling of trial activities. Telemedicine visits can be adapted to the needs of participants and the availability of health professionals, with the possibility of connecting several health professionals to the same telemedicine visit. In addition, the ability to include family and caregivers in the calls extends the spectrum of patient support and understanding of the study procedures, and can also help clarify follow-up questions without the need to travel. And lastly, the ability to conduct these visits in locations other than the participant’s home can increase the comfort and convenience of the process, allowing for greater participation and reducing waiting times.

However, these advantages are countered by several weaknesses inherent to the use of telemedicine in clinical trials, many of them overlapping with other processes such as the implementation of decentralised/remote (electronic) consent or remote screening. Disparities in internet access may limit the participation of certain demographic groups, leading to inequities in the representativeness of the included participants. In addition, any failures in the internet connection or devices used may hinder effective communication between the patient and the health professional, compromising the quality of the information recorded. This is added to the lack of in-person physical screening (if not performed during home health visits), which may result in the omission of underlying conditions that are not evident through videoconferencing. Videoconferencing can be burdensome for the research team if not already in use in standard clinical practice. On the other hand, problems related to privacy persist and include difficulties in verifying identity and protection of the data collected. Some difficulties derive from the remote interaction itself, such as not knowing if there are other people in the room and guaranteeing medical confidentiality.

No additional ethical, legal or regulatory factors were identified that could facilitate or enhance the implementation of telemedicine visits in DCTs (opportunities).

Finally, there are threats that may compromise the effectiveness of telemedicine visits in decentralised clinical trials. The lack of experience, expertise, guidelines, and evidence on how ethics committees should adequately assess this and other elements of DCTs represents a major threat, as it may limit the ability to implement best practices and ensure the safety and reliability of the data collected. Moreover, legislative barriers at the national level may create additional obstacles and territorial differences in the implementation of telemedicine, underscoring the need for a clear and consistent regulatory framework for its application in the context of clinical research.

### KTA5. Participant self-monitoring

Self-monitoring has several strengths. One of the main advantages lies in the reduction of participant burden, facilitated by devices that allow continuous, non-invasive monitoring of study-relevant parameters. This capability not only enhances the participant’s experience, but can also generate real-world data (RWD), enriching the understanding of the effects of an investigational medicinal product (IMP) or an intervention in everyday conditions. In addition, self-monitoring can potentially prevent adverse events by providing alarm systems that alert the participant before the onset of potentially dangerous symptoms, enabling a faster and more effective response by the clinical team. The autonomy of the participant is strengthened through the use of devices that allow them to have greater control over their own health and treatment. This autonomy, combined with the use of electronic patient reported outcomes (e-PROs), would not only improve the quality of the data collected (RWD), but also encourage greater participation and engagement in the study.

However, self-monitoring also has weaknesses that need to be addressed; identifying device failures can be difficult, which could compromise data integrity and participant safety. In addition, dependence on electricity and internet connectivity raises concerns about data integrity, equity of access to technology, especially for isolated, marginalised or resource-limited populations. Excessive data generation and the lack of validated processes for data analysis can make it difficult to draw relevant conclusions and make informed clinical decisions, and place an additional burden on staff dedicated to overseeing collected data. In addition, data protection concerns related to confidentiality and safety of participant data may arise due to the storage of information on third-party servers. Finally, it is difficult to guarantee the proper use of the devices limited to the participant only.

No opportunities were identified for this KTA. Finally, there are threats that may compromise the implementation of self-monitoring. Post-trial access to the monitoring technology used during the trial presents logistical and ethical challenges in terms of accountability and patient safety. In addition, some applications and devices used may be considered as medical devices and may be affected by more restrictive regulation, which would make their acceptance and use in the context of clinical trials more difficult. Another ethical dilemma is the increased time that the participant has to spend on these activities as well as related financial compensation and its impact on their decision to participate.

### KTA6. Delivery and return of investigational product

One strength was identified with respect to the at-home delivery, as the burden on participants in the collection and return of the investigational medical product (IMP) is reduced. This reduction in travel and logistical barriers may encourage greater participation and retention of participants.

Weaknesses that need to be addressed to mitigate the risks associated with the delivery and return of IMP in decentralised settings include concerns about compliance with Good Clinical Practice (GCP) and Good Manufacturing Practice (GMP). Ensuring evidence of receipt of IMP is critical, as is ensuring proper storage and handling to maintain the integrity and quality of the product. The potential loss of pharmaceutical supervision also raises concerns in terms of product safety and quality, which underlines the importance of implementing adequate control and monitoring measures. In addition, concerns about the protection of participant information, especially regarding data sharing with vendors (e.g. the shipping company with which the shipment/pick-up is made) should be addressed by implementing clear security measures and protocols to protect the confidentiality and privacy of participant information.

No opportunities were identified in relation to this key activity. Finally, potential threats include concerns about GCP compliance in terms of evidence of receipt of the IMP, which could affect the validity and acceptability of the study. This aspect was also identified as a weakness, which shows its consideration as an internal and external aspect to the DCTs.

### KTA7. Clinical trial oversight

Clinical trial oversight, in both decentralised and conventional settings, plays a critical role in ensuring the integrity, safety, and quality of the data generated during the study. However, no aspects of DCTs were identified that could facilitate or enhance compliance with ethical, legal, regulatory and operational requirements compared to CCTs (strengths).

Among the weaknesses identified is the potential lack of medical supervision, which can make it difficult to build a strong relationship between the investigator and participants. Moreover, there is a transfer of responsibility to the participant to report adverse events, which presents potential risks to the safety and well-being of the participant. In addition, the complexity and burden of responsibility for the investigator may increase due to the need to supervise and coordinate the multiple third parties involved.

No specific opportunities in the context of decentralised clinical trial oversight were identified. In terms of threats, the complexity of third-party certification schemes is highlighted, which can make it difficult to select and monitor external providers involved in the study. This complexity may create additional challenges in terms of regulatory compliance and quality assurance when evaluating and selecting external suppliers.

### KTA8. Remote safety monitoring

Remote safety monitoring in decentralised clinical trials (DCT) plays a crucial role in the early detection of adverse events (AEs) and the continuous monitoring of participants’ well-being. One of the main strengths of this process lies in the ability to detect AEs early, allowing for rapid and effective intervention to mitigate potential health risks to participants. In addition, real-time monitoring may allow for the detection of different biomarkers before the participant presents symptoms.

However, remote safety monitoring also has weaknesses. There are concerns about the possible replacement of personal context with the exclusive use of electronic devices in monitoring, which could limit the full understanding of the participant’s health and well-being. In addition, it is important to note that remote monitoring may not be applicable to all clinical trials, which raises challenges in terms of implementation and adaptation to different settings, clinical trial types, and therapeutic areas.

No opportunities and threats were identified in the context of remote safety monitoring in this SWOT matrix.

### General SWOT analysis of DCTs

The SWOT analysis of Decentralized Clinical Trials (DCTs), compiled from individual assessments across KTAs. This resulted in a matrix with 11 strengths, 13 weaknesses, 2 opportunities and 4 threats (Table 4). DCTs are particularly strong in leveraging digital technologies to streamline processes like electronic consent, improve participant autonomy, and enhance access and data collection, especially across diverse and remote populations. They also support better monitoring and may contribute to improving participants’ digital and health literacy. However, several weaknesses were identified, including technological barriers, reduced personal interaction, privacy concerns, and logistical challenges in managing trial materials. There are also concerns about data quality and the potential loss of human connection. Opportunities lie in regulatory harmonization and broader acceptance of digital tools, while threats include fragmented legislation, limited regulatory experience with DCTs, and inconsistencies in professional standards across countries.

**Table 4:** Components of the SWOT matrix of DCT approaches

Strengths (*n* = 11)
S1. Potential benefits of using e-Consent
S2. Potential benefits of using digital technologies for other study procedures different to e-consent
S3. Remote access
S4. Greater freedom decision-making
S5. Easier to do some follow-up actions
S6. Less burden for participants
S7. Easier to detect external factors that would go unnoticed in visits performed on site
S8. Reach more geographically dispersed and diverse populations
S9. Improvement of participants’ health and digital literacy skills
S10. Increasing the data collected for both research and safety monitoring
S11. Easier to monitor/audit/inspect
*Weaknesses (n = 13)*
W1. Barriers due to use of digital technologies
W2. Lack of in-person (on-site) contact
W3. Difficulties to verify the identity
W4. Privacy issues
W5. Not suitable for all the CTs, therapeutic areas, participants and activities
W6. Increased burden and responsibility on participants to do some activities themselves
W7. More burden or risk-taking for the health care providers
W8. Difficulties in the management and organization configuration of the investigator team
W9. Difficulties in the management and conservation of the biosamples
W10. Difficulties in the management, conservation and administration of the IMP
W11. Generation of unnecessary data for the research
W12. Risk of generating invalid data or with questionable quality
W13. Dehumanisation of the participant (losing contact and feeling towards the participant by not interacting with him/her directly)
*Opportunities (n = 2)*
O1. Harmonisation in the regulation and legislation on DCTs
O2. Collaboration with local resources
*Threats (n = 4)*
T1. Non harmonization of the legislation
T2. Lack of specific knowledge and accumulated experience for ethical, legal and regulatory assessment of DCTs
T3. Regulatory requirements due to the use of multiple medical devices in DCTs (such as applications and devices used in DCTs)
T4. Professional certifications and qualifications are not homogeneous among countries.

## Discussion

The aim of this article was to describe the results of a SWOT (Strengths, Weaknesses, Opportunities, and Threats) analysis of Decentralised Clinical Trials (DCTs) from ethical, legal, regulatory, and operational perspectives. The analysis was conducted by identifying eight key trial activities (KTAs) through a qualitative study involving ten experts, resulting in a consolidated SWOT matrix comprising 31 elements. One key finding was that the most recurrent and prominent challenges identified in the analysis related to the use of digital technologies, the lack of in-person contact, and the harmonisation of regulations. Another significant finding was that, as the study was carried out in April 2022, the prevailing lack of consensus regarding DCTs was reflected in the resulting matrix: internal components (strengths and weaknesses) were more numerous and specific than external ones (opportunities and threats), which tended to be more general in nature.

### Strengths

The strengths identified in this study, such as the use of digital technologies to streamline processes, enhance participant autonomy, and improve access, are consistent with previous literature emphasizing the transformative role of digitalization in clinical research. Several authors have highlighted how wearables, telemedicine, and electronic consent contribute to greater flexibility and participant engagement [25–27]. Similarly, the FDA’s encouragement of mobile-based adverse event reporting [25] reflects a broader trend toward integrating digital tools into trial workflows. These findings are consistent with suggestions that digital technologies may drive DCT adoption [26, 27], particularly in improving monitoring and fostering digital and health literacy among participants [28].

### Weaknesses

The weaknesses observed—technological barriers, reduced personal interaction, privacy concerns, and logistical challenges—are widely documented in the literature. Studies have noted that the intensive use of technology in DCTs transfers both the advantages and limitations of these tools to the trial context [29]. Several authors [30–33] underscore the ethical and practical implications of diminished face-to-face interaction, which can hinder trust-building and individualized care. Furthermore, concerns about data quality and participant burden, particularly when tasks traditionally performed by research staff are delegated to participants or third parties, have been repeatedly emphasized [30, 34]. These issues are compounded by persistent digital divides [35, 36], which risk excluding certain populations and thereby compromising equity in trial participation [27, 31, 37] between populations both within and between countries [38].

### Opportunities

Opportunities identified in this study, such as regulatory harmonization and the growing acceptance of digital tools, are echoed in recent developments within the regulatory landscape. The issuance of updated guidelines by the FDA and EMA [8, 9, 39, 40] and the revision of ICH GCP E6 (R3) [41] illustrate an emerging trend toward formalizing decentralized approaches.

### Threats

The threats identified—fragmented legislation, limited regulatory experience, and inconsistent professional standards—are consistent with concerns raised in prior research. Despite recent progress, significant heterogeneity persists both between and within countries [11, 34], creating uncertainty for sponsors and investigators. Vayena et al. [30] and de Jong et al. [42] highlight the operational and ethical challenges arising from unclear responsibilities and varying interpretations of decentralization requirements. Additionally, the literature warns of technology-related risks such as obsolescence, which could jeopardize long-term data storage and compliance [26].

### Implications for practice and policy

The findings of this study have several implications for the future implementation and regulation of DCTs. First, the lack of harmonized regulations and standards remains a significant barrier. The development of the European Health Data Space [43] represents a promising step toward greater regulatory alignment.

From a practical standpoint, the integration of digital tools into clinical trials requires careful planning, particularly in terms of participant support, data management, and privacy protection. The study highlights the need for privacy impact assessments, especially when involving vulnerable populations, and suggests that technologies like blockchain could help mitigate data security risks [44, 45]. Moreover, the ethical principle of data minimization [18] must guide decisions about what data to collect and how it will be used [30].

The evolving composition of research teams—potentially including engineers and IT specialists—calls for a redefinition of roles and responsibilities [46]. This shift also necessitates new training and governance structures to manage decentralized tasks effectively. Collaboration with local resources, such as pharmacies or direct-to-consumer services, may offer practical solutions to logistical challenges [33, 42].

Finally, ensuring a positive participant experience is critical. As noted by Apostolaros et al. [33], even if digital interactions are convenient, the overall success of a DCT depends on the participant’s experience with all aspects of the trial, including the investigational product. Addressing these practical and ethical considerations will be essential to making DCTs a sustainable and equitable model for future clinical research.

The transition toward decentralised models is also expected to influence the cost distribution of clinical research [26]. While certain expenses—such as those related to physical site operations or participant travel—may decrease, new investments in digital infrastructure, data management, and workforce training will emerge. Building capacity through targeted training programmes will be essential to ensure that research teams can operate effectively in digitally enabled environments. Expanding practices such as home health visits could not only support participant retention and data quality but also contribute to workforce upskilling and the integration of clinical research within community healthcare structures. These shifts highlight the need for adaptive funding mechanisms and long-term planning to sustain the scalability and equity of DCT implementation across diverse healthcare settings.

### Strengths and limitations

This study presents several strengths. First, it provides a comprehensive SWOT analysis of Decentralised Clinical Trials (DCTs), covering ethical, legal, regulatory, and operational perspectives. The analysis was informed by a multidisciplinary team of experts, which enhances the depth and breadth of the insights. Additionally, the study addresses timely and relevant issues, such as the evolving regulatory landscape and the integration of digital technologies in clinical research, offering valuable guidance for stakeholders.

However, the study also has limitations. The analysis is primarily qualitative and based on expert opinion, which may limit the generalizability of the findings. Furthermore, the rapidly changing regulatory and technological context means that some observations may become outdated as new guidelines and innovations emerge. Finally, the study does not include empirical validation of the identified strengths, weaknesses, opportunities, and threats, which could be addressed in future research.

## Conclusions

Decentralised Clinical Trials (DCTs) represent more than an operational shift; they may signal a structural transformation in how clinical research is conceived and executed. While our SWOT analysis underscores both the promise and the complexity of these models, the broader outlook suggests that DCTs will increasingly shape the future of evidence generation. Their potential to enhance inclusivity, leverage real-world data, and integrate digital health innovations positions them as a cornerstone of patient-centric research. However, realising this potential requires moving beyond technical feasibility toward systemic readiness—harmonised regulations, robust governance frameworks, and sustainable infrastructures. In addition, issues of data harmonisation across platforms and the ownership of patient-generated data by Digital Health Technology developers further complicate the integration of these technologies into trial and healthcare ecosystems.

The next decade will likely witness hybrid models becoming the dominant paradigm, blending decentralised elements with traditional oversight to balance flexibility and rigour. This evolution will demand new competencies within research teams, including digital literacy, data science expertise, and adaptive regulatory strategies. Ethical considerations—such as equity in access, data privacy, and participant autonomy—must remain central to this transformation, ensuring that technological progress does not exacerbate disparities. These technological advances not only reinforce the strengths of DCTs but may also generate spill-over effects, fostering their eventual integration into conventional healthcare, particularly in the field of remote patient monitoring.

Future research should prioritise comparative effectiveness studies of hybrid versus fully decentralised designs, explore scalable solutions for data integrity and cybersecurity, and develop frameworks for global regulatory convergence. Ultimately, the success of DCTs will depend on fostering trust among stakeholders—patients, investigators, regulators, and industry—through transparency, co-creation, and continuous evaluation. By embracing these challenges as opportunities, the clinical research ecosystem can transition toward a model that is not only more efficient but also more inclusive, resilient, and ethically robust.

## Supplementary Information

Supplementary Material 1.

Supplementary Material 2.

Supplementary Material 3.

## Data Availability

Data is provided within the manuscript or supplementary information files.

## Footnotes

1 Taking into account the criteria of Kennedy and Price, quoted in Yañez et al. [24], we considered as experts those who can make relevant contributions, given their knowledge or experience.

## Abbreviations

AE: Adverse Event
CCT: Conventional Clinical Trial
CT: Clinical Trial
CTTI: Clinical Trials Transformation Initiative
DCT: Decentralised Clinical Trial
EAGLE: Ethics, regulAtion, Gcp, LEgal and data privacy aspects
e-Consent: electronic Consent
EMA: European Medicines Agency
ePRO: electronic Patient-Reported Outcomes
FDA: Food and Drug Administration
GCP: Good Clinical Practice
GDPR: General Data Protection Regulation
GMP: Good Manufacturing Practice
HMA: Heads of Medicines Agencies
HTA: Health Technology Assessment
ICH: International Council for Harmonisation
IMI: Innovative Medicines Initiative
IMP: Investigational Medical Product
KTA: Key Trial Activity
SWOT: Strengths, Weaknesses, Opportunities and Threats
