# Supplementary material for: SWOT analysis of decentralised clinical trials from an ethical, legal, regulatory and operational perspective
Source: BMC Med Ethics. 2026 Feb 9;27:57. doi: 10.1186/s12910-026-01391-w (PMC12990590; doi:10.1186/s12910-026-01391-w)
Supplement: Supplementary file 1 — Supplementary Material 1. [file 12910_2026_1391_MOESM1_ESM.docx]

**Additional file 1: Description of the Units of Analysis:**

| Unit of analysis | Description of the activity |
| --- | --- |
| Implementation of decentralised/ remote (electronic) consent | In traditional clinical trials, during the informed consent process, the potential participant receives the study information sheet, which includes, among other aspects, the justification and objectives, procedures, potential risks and benefits, and treatment alternatives. Participants should be given as much time as needed to read it.  Subsequently, a face-to-face interview with the investigator takes place, in which the information is explained to the potential participant, it is verified that he/she has understood it. Participants should be given sufficient time and opportunity to address concerns, ask questions and receive answers before (and during) the trial.  Finally, the informed consent form is signed by both the participant and the investigator.  If this procedure takes place remotely, the informed consent will usually be in electronic format including: the verification of the potential participant's identity, the study information, the interview in a telemedicine visit and the electronic signature. |
| Decentralised screening of potential trial participants | In traditional clinical trials, after signing the informed consent form in person, the investigator verifies and confirms again that the participant meets the criteria for participation by interviewing him/her and reviewing his/her medical history. Depending on the requirements of the protocol, a physical examination is performed, measurements of vital signs and relevant tests are performed.  If this procedure takes place remotely through a telemedicine consultation, it is necessary to establish how the investigator will obtain the relevant information from the participant's medical history.  As for the physical examination and assessment of vital signs, depending on the study protocol and the electronic devices available, the participant himself may perform certain procedures and send the results to the investigator.  It is also possible for a health professional to come to the participant's home to perform the physical examination or ask the participant to visit a local family doctor to perform a complete physical examination and then send the report to the investigator. |
| Home health visits | In traditional clinical trials the participant travels to the trial site where the clinical trial takes place.  In a decentralised trial, certain procedures can be performed remote form site (e.g. at home or place of work) by a health professional, doctor or nurse, thus avoiding the need for the participant to travel to the clinic or site. |
| Telemedicine visits | In traditional clinical trials, most visits are conducted face-to-face at the trial site. Occasionally follow-up telephone calls may be made to obtain clinical trial safety data.  In telemedicine visits, the participant contacts a member of the research team (or vice-versa) via a videoconferencing system. |
| Self-monitoring | In traditional clinical trials, the required measurements and tests are performed in person at the trial site by healthcare professionals.  In a decentralised trial, the participant will be able to perform the measurements and tests remote form site (e.g. at home or place of work), sometimes continuously if a device allows it. Depending on the technology, the volume of data generated may be greater and provided at more regular intervals than in the traditional mode. |
| Delivery and return of investigational product | In traditional clinical trials, the investigator team either administers the IMP in the trial site or provides it for the participant to take at home according to the established schedule. At the end of the treatment period, any unused investigational product is returned by the trial site to the sponsor or destroyed locally according to the standard operating procedure.  In remote clinical trials, there is the possibility that the research team sends the medication to the participant's home, always with complete instructions and training regarding storage, administration schedule, and return conditions. |
| Clinical trial oversight | In traditional clinical trials, all the clinical trial procedures are carried out at the trial site, so there is proximity and direct contact between the members of the research team and the participant.  In decentralised trials, some procedures are performed remote from the trial site (e.g. at the participant's home), either by the participant or by third parties who are not direct members of the research team (e.g., companies with home health nursing services), hence a different work dynamic and professional relationship will be established. |
| Remote safety monitoring | In traditional clinical trials, safety data are obtained in several ways: by review of the participant´s medical history by the investigator at the times established according to the protocol (which may or may not coincide with the on-site visits at the trial site), through information recorded by the participants in the logbook-diaries provided for this purpose (that will be reviewed by investigator in the next visit at the trial site), by interview with the participants during the on-site visits at the trial site and by spontaneous notification by the participant (if he/she contacts the research team about an event that he/she considers important or is concerned about and according to the training in adverse event reporting that he/she received at each on-site visit).  In decentralised trials, a participant may still use logbooks / diaries, however, the procedure may be different in that the receipt of safety data may be continuous. Sometimes, the investigator may even receive an alert for an abnormal value in a certain parameter before the participant has objectified it or noticed any symptoms. |
