# Supplementary material for: SWOT analysis of decentralised clinical trials from an ethical, legal, regulatory and operational perspective
Source: BMC Med Ethics. 2026 Feb 9;27:57. doi: 10.1186/s12910-026-01391-w (PMC12990590; doi:10.1186/s12910-026-01391-w)
Supplement: Supplementary file 2 — Supplementary Material 2. [file 12910_2026_1391_MOESM2_ESM.docx]

**KTA1. Implementation of decentralised/ remote (electronic) consent**

| Strengths |
| --- |
| - Adjust to literacy of potential participants - Autonomy: easier to withdraw from the study - Better informed participants through digital means (e.g., video/audio) - Easier to include participants (no travel) - Easier to store/manage/document the informed consent (easier to monitor/inspect) - Facilitate increased health literacy (by more easy explanation of health terms) - Improved recruitment (and retention because they understand better what they sign up for) - Include a quiz to guide IC discussion and check participant understanding - Keeping participants involved during and after trial (e.g., sharing results) - More diverse populations (opportunity to include people from any part of the territory). - More user-friendly forms – increased satisfaction - Opportunities for re-consenting and providing extra information (e.g., substantial amendment) - Opportunity to put layers of information (additional information if wanted) - Participants can do more an independent decision (because no pressure from doctor) - Participants can easily go back to information/access to information - Standardize information |
| Weaknesses |
| - Costs of the developing electronic consent - Difficult to check/test understanding - Exclusion of digital illiterate participants, participants without internet connection or access to connected devices (costs of electricity) - Missing information due to layering - Missing non-verbal communication - No face to face contact – trust building/building rapport - No paper backups (not specific for DCTs) - Risk of focusing consent only on digital materials and de-emphasising the importance of discussion - Verification of identity is more difficult - Verify voluntary consent is more difficult - You don’t know if there are others in the room during IC discussion |
| Opportunities |
| - Development and implementation of applications for eSignature/ID verification - Development of applications for eSignature/ID verification - Harmonized regulations regarding remote consent and authorisation of eIDs/eSignature |
| Threats |
| - Digital/electronic signatures not acceptable/allowed - Legislation not harmonized/lacking |

**KTA2. Decentralised screening of potential trial participants**

| Strengths : |
| --- |
| 1. Easier scheduling 2. Less burden for a selected group of people 3. Less burden for the participants (no travel to the site) 4. Less travel burden for IMP administration 5. Participant would feel more comfortable in their own environment 6. Possibility to collect information from the participants' environment that would not be captured on site. 7. Recording the examination can lead to better diagnosis 8. Suitability done by experts without being a burden for the participant (no travel time) |
| Weaknesses: |
| 1. Bad storage of recordings (privacy issues) 2. Incorrect measurements done by the patients (use of technology and devices) 3. It is more difficult to build a relationship of trust between the participant and the doctor 4. Lack of physical examination (just interview) 5. More burden for a selected group of people 6. Noisy background difficulty the interview 7. Participant feeling uncomfortable about recording their physical examination (participant does not know who is on the other side of the camera) 8. Privacy aspects (are we invading the privacy of the person's home?) 9. Recording of personal information without consent 10. Researcher verification (the participant might not be able to verify the id of the researcher) 11. Safety aspects (do we see what we want to see?) 12. Unable to verify underlying conditions that would make the participant not suitable for CT (e.g. alcoholism, physical characteristics not visible by video) |
| Opportunities: |
| 1. Participants autonomy increased due to the flexibility or have a say on different approaches 2. Potential solution: physical examination done by local GP |
| Threats: |
|  |

**KTA3. Home health visits**

| Strengths |
| --- |
| 1. Being able to detect other external factors that would not be registered during a clinic visit 2. Comfortable for some participants 3. Easier to detect ancillary care obligations (extra need of care, environment might be risky for the participant, finding child abuse) 4. Less travel burden (e.g. elderly, children, mobility issues) 5. Possibility to have the visit in a different place from home |
| Weaknesses |
| 1. Biosamples transportation might be risky (temperature) 2. Different levels of nurse certification (not all the nurses are allowed to perform all the procedures) 3. Not all the actions can be performed due to the product characteristics (vaccines, phase I trials) 4. Oversight issues (third parties performing the visits) 5. Participant might be stressed by the visit of a nurse 6. Risk of contamination due to home environment 7. Samples might be ruined by external factors or bad management 8. Shortage of qualified health care professionals who can perform home health visits 9. Some PIs would not be willing to delegate activities to the nurses 10. They can be perceived as invasive (neighbors know, might think that participant is ill, stigma) |
| Opportunities |
|  |
| Threats |
| 1. Barriers to conduct multi-country trial (local regulations) 2. Different levels of nurse certification (not all the nurses are allowed to perform all the procedures) 3. Regulators might not have a clear picture of qualifications for the person who performs the home health visit 4. Shortage of qualified health care professionals who can perform home health visits |

**KTA4. Telemedicine visits**

| Strengths |
| --- |
| 1. Caregivers might be included in the call or follow-up instructions 2. Easier inclusion of participants 3. Flexible scheduling 4. Good for follow-up visits (clarification of question that the participant might have) 5. Less burden (travel time) 6. No waiting times at sites 7. Possibility to have the visit in a different place from home 8. Possibility to include several professionals in the same call |
| Weaknesses |
| 1. Burden for the investigator team if increase frequency of unscheduled telemedicine visits 2. Data protection 3. Digital exclusion 4. Inequality on having internet connection 5. Internet connection or devices failure might difficult the consult 6. Lack of physical examination 7. Medical confidentiality 8. Privacy issues (you don't know if there are others in the room) 9. Recording might be done wrong (lack of SOPs) 10. Risk of missing underlying conditions that might not be detected by video 11. Verification of identity |
| Opportunities |
|  |
| Threats |
| 1. Lack of experience, expertise, guidance, habits and evidence on how to assess this and other DCT elements 2. Legislation at the national level barriers and make a difference on the implementation of telemedicine |

**KTA5. Self-monitoring**

| Strengths |
| --- |
| 1. Improve digital literacy for health improvement 2. Less participant burden (devices that make continuous monitoring) 3. Participant autonomy 4. Post-trial access (better adherence to treatment after trial) 5. Potential Real World Evidence gathering 6. Prevention of AE, alarm system that might alert the patient to seek help before having symptoms 7. Thresholds that trigger response (for faster reactions in case of safety issues) 8. Use of electronic Patient Reported Outcomes (e-PROs) |
| Weaknesses |
| 1. Burden on responsibilities performing the tasks or performing the monitoring 2. Data protection issues (data might be stored at vendors servers) 3. Data waste (too much necessary or unnecessary data, data overload, lack of validated process on how to draw inferences/analysis) 4. Difficult to identify devices failure 5. Electricity, internet, reimbursement 6. Participant identification (somebody else can use the device, or collect other data) 7. Surveillance issues |
| Opportunities |
|  |
| Threats |
| - Over regulation (use of apps, devices might be overused on DCTs) - Post-trial access (after trial access to device, how to deal with that?) - Time from participant (reimbursement) |

**KTA6. Delivery and return of investigational product**

| Strengths |
| --- |
| 1. Low burden for patients to pick-up the IMP (travel time, mobility) |
| Weaknesses |
| 1. Data integrity issues 2. Data protection concerns (sharing participant data with subcontractors) 3. GCP concerns (proof of receipt, how to deal with it is a regulatory requirement) 4. GMP concern (IMP temperature, etc.) 5. Losing pharmacist oversight (safety, and quality of the product) |
| Opportunities |
|  |
| Threats |
| GCP concerns (proof of receipt, how to deal with it is a regulatory requirement) |

**KTA7. Clinical trial oversight**

| Strengths |
| --- |
|  |
| Weaknesses |
| 1. High burden of responsibility for the PI (more third parties to take care of) 2. PI responsibility complexity 3. Potential lack of medical context/difficult to build a relationship with the PI 4. Transfer of responsibility to the participant might lead to risk of no detecting adverse events |
| Opportunities |
|  |
| Threats |
| 1. Complexity of certification schemes for third parties |

**KTA8. Remote safety monitoring**

| Strengths |
| --- |
| 1. Early detection of AEs 2. Real time monitoring detecting different biomarkers before the participant shows symptoms (lifting burden from the participants) |
| Weaknesses |
| 1. Concerns that might substitute on person context and rely on only devices 2. Not applicable to all the trial (also applicable to other DCT activities) |
| Opportunities |
|  |
| Threats |
|  |
